# Outcome-adaptive randomization in clinical trials: issues of participant welfare and autonomy

**DOI:** 10.1007/s11017-019-09481-0

**Published:** 2019-02-18

**Authors:** Julius Sim

**Affiliations:** Institute for Primary Care and Health Sciences, Keele University, Staffordshire, ST5 5BG UK

**Keywords:** Outcome-adaptive randomization, Clinical trials, Ethics, Equipoise, Consent

## Abstract

Outcome-adaptive randomization (OAR) has been proposed as a corrective to certain ethical difficulties inherent in the traditional randomized clinical trial (RCT) using fixed-ratio randomization. In particular, it has been suggested that OAR redresses the balance between individual and collective ethics in favour of the former. In this paper, I examine issues of welfare and autonomy arising in relation to OAR. A central issue in discussions of welfare in OAR is equipoise, and the moral status of OAR is crucially influenced by the way in which this concept is construed. If OAR is based on a model of equipoise that demands strict indifference between competing interventions throughout the trial, such equipoise is disturbed by accruing data favouring one treatment over another; OAR seeks to redress this by weighting randomization to the seemingly superior treatment. However, this is a partial response, as patients continue to be allocated to the inferior therapy. Moreover, it rests upon considerations of aggregate harms and benefits, and does not therefore uphold individual ethics. Issues of fairness also arise, as early and late enrollees are randomized on a different basis. Fixed-ratio randomization represents a fuller and more consistent response to a loss of equipoise, as so construed. With regard to consent, the complexity of OAR poses challenges to adequate disclosure and comprehension. Additionally, OAR does not offer a remedy to the therapeutic misconception—participants’ tendency to attribute treatment allocation in an RCT to individual clinical judgments, rather than to scientific considerations—and, if anything, accentuates rather than alleviates this misconception. In relation to these issues, OAR fails to offer ethical advantages over fixed-ratio randomization. More broadly, the ethical basis of OAR can be seen to lie more in collective than in individual ethics, and overall it fares worse in this territory than fixed-ratio randomization.

## Introduction

Recent advances in randomized clinical trials (RCTs) include the use of adaptive designs. Such studies incorporate changes to trial design as the study proceeds, including changes to randomization [1]. Covariate-adaptive randomization, for example, modifies allocation to achieve optimum balance between groups on baseline characteristics. Outcome-adaptive randomization (OAR) also adjusts the allocation of participants, but on the basis of accruing outcome data—provided that such data are available in a suitably timely manner—such that participants are allocated with greater probability to the treatment that hitherto appears to be superior. In a traditional RCT, however, randomization is in a fixed ratio (usually 1:1). This allocation persists throughout the duration of the trial, unless a planned interim analysis leads to its early termination or to an arm being dropped prior to the end of the study. Allocation of participants in traditional RCTs is therefore independent of any accruing data, in contrast to the dynamic method of randomization used within OAR.

There is a broad literature on the methodological and statistical aspects of OAR. More recently, a number of articles have addressed some of its ethical implications [2–13]. This paper seeks to contribute to this discussion in the context of a simple two-arm RCT,^1^ with a particular focus on two central ethical issues: welfare and autonomy. I argue that OAR faces challenges in relation to each of these issues.

### Protecting participant welfare

A method that minimizes the allocation of participants to a putatively inferior treatment appears to be ethically advantageous. In particular, it has been suggested that OAR has the merit of favouring the notion of *individual*, as opposed to *collective*, ethics [7, 18–21]—a distinction developed in the specific context of clinical trials by Joseph Lellouch and Daniel Schwartz:

Un schéma expérimental ou une stratégie sont basés sur l’éthique collective s’ils permettent de maximiser le “bénéfice” total du groupe et qu’ils se fondent au contraire sur l’éthique individuelle, s’ils permettent de maximiser le “bénéfice” de chaque sujet, pris individuellement, au moment où on a à le traiter. [22, p. 128]^2^

Broadly, therefore, collective ethics justifies actions in terms of their aggregate benefit or reduction of harm. Clinical research can thereby be morally justified on the basis of the benefits that are expected to flow to future patients or to the population at large. On this way of thinking, such benefits, owing to their larger scale, may justifiably outweigh any harm or loss of benefit that may arise in respect of the smaller number of individual participants within the study, provided that such harm or loss of benefit is minimized. Individual ethics, on the other hand, places emphasis on the welfare of the individual. In particular, it resists subjugation of the individual’s interests to those of a broader collectivity. The moral reasoning underlying collective ethics is fundamentally consequentialist, whereby the criterion of right action is ultimately the aggregate balance of harms and benefits. In contrast, the reasoning underlying individual ethics is closer to deontology, in which the criterion of right action is centred on appraising benefits and harms in relation to specific individuals. Deontology insists that moral decision-making should take account of the ‘distinction between persons’ [23, p. 134] and should not analyse harms and benefits solely at an aggregate level. The distinction between collective and individual ethics is often mapped onto that between the role of researcher and the role of clinician, respectively [24–27].

Importantly, Lellouch and Schwartz [22], and others [24, 27–29], interpret individual ethics literally, in terms of the individual patient. A different interpretation is to contrast individual and collective ethics in terms of ‘doing what it best for current subjects in the trial versus doing what is best for future patients’ [21, p. 174]. This suggests not so much a contrast between the individual and the collectivity as one between two collectivities—one (smaller) consisting of the patients in the trial and another (larger) consisting of future patients who stand to benefit from the results of the trial. However, the more plausible construal of individual ethics—to be adopted here—is in terms of the welfare of each person, taken singly.

### Equipoise

A principle in the ethics of RCTs that is advanced in support of individual ethics is equipoise. There are somewhat different interpretations of the principle [30], but they have in common the notion that random allocation to the interventions tested within an RCT is ethically justified if there is uncertainty as to their relative effectiveness. As originally formulated by Charles Fried [31], what has come to be known as ‘theoretical’ (or ‘individual’) equipoise requires that the individual investigator be indifferent as to the relative effectiveness of the interventions. Subsequently, Benjamin Freedman [32] developed a form of ‘clinical’ (or ‘community’) equipoise that locates this uncertainty at the level of the clinical community, such that what matters ethically is that there is indifference among clinicians in general as to the optimum treatment, regardless of whether individual practitioners have treatment preferences. Crucially, if the demands of equipoise (on either definition) are satisfied, patients are not knowingly disadvantaged by being randomized to one arm of the trial rather than another. Nicolas Fillion [13] points out that an additional requirement on a trial is that it should be capable of disturbing—or at least contributing to disturbing—the state of equipoise that existed at the outset; so equipoise must exist, but it must also be assailable.

### Two models of equipoise in outcome-adaptive randomization

Equipoise has been discussed by several authors in specific relation to OAR [2, 9–13]. However, an analysis of the ethics of OAR depends crucially on how equipoise is conceived. The distinction between theoretical and clinical equipoise outlined above is based on where equipoise is judged—at the level of the individual investigator or at that of the clinical community. A more pressing concern for the ethics of OAR, however, is how emerging evidence is acted upon in relation to equipoise. A somewhat different distinction is therefore required between two models of equipoise. On one reading, which I refer to as E1, data emerging from the trial that appear to favour one intervention over another serve to disturb the state of equipoise immediately and thereby create a correspondingly immediate moral imperative to increase the probability that participants will be allocated to the better-performing intervention. Thus, Scott Saxman notes:

Outcome-adaptive randomized trials start out in equipoise, but equipoise is disturbed as soon as data are available from the first group of patients enrolled into the study and the randomization is adapted to favor the ‘better’ treatment arm. [9, p. 63]

The second reading of equipoise, which I label E2, is offered by Alex London [11] and Laura Bothwell and Aaron Kesselheim [12], who advance a different relationship between emerging data and the adjustment of randomization weights from that proposed within E1. London argues that OAR is compatible with clinical equipoise because the latter does not require that randomization probabilities should be equal:

If it is consistent with concern for welfare for a patient to be directly treated with A or B or C (to receive that intervention with certainty), then it cannot violate concern for welfare if that patient is assigned to those interventions with any distribution of probabilities that sums to 1. [11, p. 412]

This is persuasive. If *k* is the number of treatments under test, it does not matter ethically that some participants are randomized to treatment A with a probability less than 1/*k*, since treatment A is regarded as optimum by a portion of the expert community (even if the other treatments under test are preferred by a larger portion of the community). It is common for trials using fixed-ratio randomization (FRR) to employ unbalanced randomization in order to gather fuller information on one treatment than another, or because access to one treatment is more limited than access to another, or because doing so secures greater statistical power in certain multi-arm trials [33].^3^ In terms of London’s argument, this practice is acceptable.

However, when one considers OAR, the issue is not simply the presence of unequal randomization weights, but their adjustment—and specifically, their adjustment on the grounds of participant welfare. London maintains that OAR is consistent with clinical equipoise because:

even if rational inquirers recognise that initial evidence from a clinical trial supports the clinical merits of one intervention (A) over the others (B or C), that evidence may not be strong enough to lead responsible experts to alter their recommendation, or to alter the recommendation of every expert in that community. [11, p. 413]

Interventions B and C therefore remain admissible treatments within the trial notwithstanding such initial evidence—their appropriateness is not questioned under E2 in the way that it would be under E1. While this argument reconciles unbalanced randomization with clinical equipoise, it is not immediately obvious how it provides a moral rationale for OAR. If an existing imbalance is compatible with clinical equipoise, what is the motivation for adjusting it in the light of accruing evidence? London’s explanation is that OAR:

should be seen as modelling an idealised health system in which diverse communities of fully informed experts who disagree about the relative merits of a set of interventions shrink or grow as their constituent members update their expert opinions in light of reliable medical evidence. In this view, randomisation weights are … an idealised representation of the probability that a patient in such an idealised learning health system would encounter a practitioner from these communities if they were to be allocated to a clinician at random. [11, p. 412]

Within E1, participant welfare depends upon the investigator’s responding continuously to accruing data, such that data favouring one intervention over another are evidence of its superiority and therefore disturb equipoise, requiring an adjustment to randomization probabilities at that juncture. In contrast, E2 does not regard such data as evidence of overall treatment superiority or inferiority, maintaining that the initial state of clinical equipoise can survive such evidence until such a point that differences in outcome ‘are sufficiently convincing to reasonably inform the medical community and clinical practice’ [12, p. 28]. In this way, no immediate change to randomization probabilities is required. Thus, within E2, participant welfare is promoted differently, by adjusting the probability of randomization to a particular intervention in proportion to the size of the clinical community favouring that intervention, such that ‘patients in [an OAR] study have a better chance of being treated with what is ultimately recognised as the best treatment for their condition’ [11, p. 413]. On this account, and in contrast to E1, changing randomization probabilities are not a direct response to emerging evidence of treatment effectiveness. Instead, these data are taken as predictive of clinicians’ behaviour in response to such evidence, and it is this anticipated change (or lack of change) in behaviour that is reflected back to motivate the adjustment of randomization probabilities.

Equipoise is clearly a more acute problem in the context of OAR under E1 than under E2; indeed, E2 obviates many of the equipoise-related concerns that arise within OAR. I do not seek here to arbitrate between these two models of equipoise in terms of their relative strengths and weaknesses, or otherwise privilege one account over the other; nor will I assess moral or epistemological challenges that have been made to the overall concept of equipoise [34–38]. Instead, I focus my discussion in the remainder of this section on the situation where, as commonly occurs, advocates of OAR base their standpoint on an interpretation of equipoise that aligns with E1, and I explore the challenges that such an account faces when viewed on its own terms.^4^

### Responding to (loss of) equipoise under E1

It is clear that equipoise is handled differently in FRR and OAR. In both cases, the trial begins in a state of equipoise. In FRR, beyond any planned interim analyses, assessments of relative treatment effectiveness are not made, and therefore equipoise is not reassessed, until completion of the trial. In OAR, treatment effectiveness is continuously reassessed, and therefore equipoise is continuously re-evaluated; according to E1, if equipoise is found to be disturbed, then allocation is adjusted in favour of the hitherto superior treatment. The consequence, however, is that participants are still randomized to the apparently inferior treatment, albeit at a lower rate. This compensates for a loss of equipoise, but it does not restore it because, for Saxman [9], the trialist is knowingly randomizing some participants to a treatment believed to be inferior.^5^ Judged in terms of E1, this is, on the face of it, ethically problematic.

The advocate of OAR might respond that because allocation is weighted towards the treatment judged to be superior, most patients will now receive the superior intervention. There are three points to note here. First, such an argument retreats from individual ethics, as it rests upon a notion of aggregate benefit—the fact that *most* participants will receive the superior treatment is taken to justify the continued allocation of a smaller group to the inferior treatment.^6^ This constitutes a consequentialist justification, reflecting the notion of collective ethics, and any attempt to appeal to individual ethics in support of OAR therefore founders.

Second, it rests on a questionable moral logic, whereby the action taken only partially fulfils the moral consideration that prompts it. Thus, Richard Royall proposes a more consistent response, arguing that ‘after finding enough evidence favoring A to require reducing the probability of B, the physician obeying the personal care principle must see that the next patient gets A, not just with high probability, but with certainty’ [40, p. 58].

Third, it depends upon what is meant by ‘most’ participants. The weighting of randomization in OAR appears to ensure that the *proportion* of participants allocated to the inferior treatment in OAR is henceforth smaller than in FRR. However, as a trial based on OAR is likely to require more participants, at given levels of statistical significance and power, than one based on FRR, the *number* of participants allocated to the seemingly inferior treatment may be greater than under FRR [5, 41, 42].^7^ Hence, it is true that within a trial OAR will normally minimize the proportion, and thus the number, of participants randomized to the inferior treatment. However, if one is considering a comparison between a trial based on OAR and one based on FRR—as I am here—then while the proportions will still favour OAR, the numbers may not. Of course, there may also be a larger number of patients allocated to the superior treatment under OAR than under FRR. This, however, would count in favour of OAR only if one were to accept a consequentialist moral calculus that permits a direct trade-off between benefits and harms—a calculus that is out of keeping with the deontological basis of individual ethics, which would place some degree of prohibition on harm even in the face of a greater countervailing benefit. Moreover, setting aside the distribution of participants across the arms of the trial, any increase in the sample size, and the associated costs of a study, raises morally relevant issues of efficiency [2, 13, 44].^8^

If one considers participants across the duration of the trial, an additional difficulty emerges in respect of participants enrolled either early or late. Turning first to those enrolled early, Edward Korn and Boris Freidlin point out that at the outset of a trial using OAR there is little information on which to base the weighting of randomization [41]. The intention to randomize preferentially to the superior treatment is therefore realized minimally, if at all, at this juncture. As such, on epistemic grounds, the proposed ethical merit of OAR cannot be claimed for those enrolled early.

Conversely, with respect to those enrolled later in the trial, as the study proceeds and data on outcomes accrue, the informational basis for OAR is augmented; and while most participants are randomized to the favoured treatment, others continue to be randomized to a treatment increasingly disfavoured by the data. In this way, some individuals enrolled late in the trial receive a treatment that is strongly disfavoured by the accumulating data, which does not uphold participant welfare under E1. Accordingly, any moral justification for allocating participants to the apparently inferior treatment becomes increasingly tenuous as the trial proceeds. So, in different but equally problematic ways, ethical difficulties occur with OAR vis-à-vis both early and late enrollees—for the former, no benefit seems to accrue through OAR in terms of welfare, and for some of the latter, a loss of such benefit is countenanced.

For Saxman [9], the fact that early and late participants have differing probabilities of receiving the ostensibly superior intervention is problematic for justice as it applies to the fair distribution of benefits and burdens. Relevant here is a consideration of procedural justice, which specifically concerns the processes and methods whereby benefits or burdens are allocated. One can note that, throughout the course of the study, OAR allocates all participants (except for the very first) with greater likelihood to the apparently superior treatment, according to the current state of knowledge at the time of enrolment. In terms of what John Rawls [45] calls pure procedural justice—whereby, once the procedure of allocation is deemed just, there is no separate criterion for judging the outcome of such allocation—there would seem to be no difficulty with OAR, since every participant is treated in a similar way, given current evidence from the trial. However, other readings of procedural justice, which Rawls calls perfect and imperfect procedural justice [45], require an independent assessment of the substantive outcome of the process of allocating benefits and burdens.^9^ On this basis, it is hard not to be uneasy at the differing prospects of benefit for early versus late enrolment within OAR among participants who are ‘otherwise equal’ [9, p. 64]. The procedure of allocation alone does not seem to provide sufficient reassurance, and independent justification of its outcome is needed.

One way to lessen this concern would be to appeal to the notion of choice. Provided that they are told how allocation probabilities may change, participants can decide for themselves what probability they find acceptable and time their enrolment accordingly. However, such choice is not available to all. Necessarily, not all participants can choose to delay enrolment in order to secure favourable probabilities, as such a decision is possible only insofar as others have already enrolled first. Additionally, it has been pointed out that, in many trials, those who are sicker or have a poorer prognosis cannot afford to delay enrolment [2], so not everyone can choose to join a study at a potentially advantageous time. Finally, concerns related to comprehension—to be addressed later—suggest that only those participants who fully understand the implications of changes in allocation probabilities would be able to exercise such choice effectively [10].^10^ Drawing on psychological research [46], Saxman suggests that there is a stronger appeal to fairness when individuals know that their outcomes differ from those of others than when they are unaware of such differences [9, p. 64]. While this notion may explain *perceptions* of fairness or unfairness, more is required to settle the issue of whether a specific distribution is intrinsically fair. Appeals to choice, or to individuals’ perceptions, seem to translate the issue into one of autonomy, leaving the justice of differing levels of benefit and burden over the course of the trial in need of justification.

In Christopher Palmer’s view, the fact that late enrollees may do better than early enrollees is ‘what medical progress is all about anyway—treating tomorrow’s patients better than today’s’ [47, p. 395]. The appeal to clinical practice does not, however, appear apposite here. Advances in medical treatment are a welcome consequence of research, but the time at which patients present for such treatment is a natural process, and hence the way in which the benefits of medical advances are distributed to patients over time is not a matter of human decision. In a trial, however, any differential distribution of therapeutic benefit arising from the design and conduct of the study is the responsibility of the investigator and requires a specific moral justification. Palmer also argues that early enrollees may be comforted by the knowledge that patients in trials tend to fare better than those outside trials [47]. This too does not seem to address the issue—the concern here is the fair treatment of individuals within a trial, not how they are treated in comparison to others outside the context of medical research.

Let me switch the focus now to the end of the trial. Steven Piantadosi states that a motivation for adaptive methods is ‘a desire to minimize the number of subjects entered on what *will be* shown to be the inferior treatment’ [48, p. 340] (emphasis added). This motivation points to a proleptic assumption about the outcome of the study that may be unwarranted. As Marc Buyse has indicated [4], the definitive conclusion reached on the treatments being tested may be at odds with the allocation that has occurred through OAR during the trial (owing to the imprecision with which the adaptive allocation probabilities are estimated from the data). Thus, the weighting of randomization throughout the trial at times may be in the direction of the inferior treatment, with the result that most participants will have received this ultimately disfavoured intervention [49, 50]. The moral objective of OAR is thereby wholly frustrated.

Even if OAR does weight allocation probabilities in line with the overall verdict of the study, it is important to demonstrate that it has done so on the basis of sufficient and relevant evidence. Adjustment to randomization has to be made on the basis of data that are available in a timely manner, which will normally mean a short-term outcome. If longer-term outcomes are more relevant, but are not available to form the basis of such adjustment, the informational basis for changing randomization probabilities may be incomplete (because other important information is unavailable) or unsound (because using only short-term information may not reflect a more global judgment that would be reached across all outcomes).^11^

A final ethical difficulty facing OAR is that of demonstrating why an accumulation of evidence that, within E1, is considered sufficient to disturb equipoise, and thus to justify weighting randomization against one intervention on the grounds of its perceived inferiority, is not also a reason to stop the trial altogether, as would likely occur during a planned interim analysis in a trial using FRR. As noted earlier, the trialist employing OAR seemingly makes only a partial response to information suggesting that some participants will be disadvantaged by allocation to a particular treatment. By contrast, terminating the trial seems to address such a loss of equipoise head-on. Advocates of OAR need to provide a clear, non-arbitrary criterion to distinguish between the level of information that requires *some* participants to be diverted from the inferior treatment and the level of information that requires *all* participants to be so diverted by halting the trial.

This problem does not just relate to participants at the point of randomization. A similar argument could be made regarding certain participants already in the trial. If accruing information is sufficient to weight subsequent allocation towards the apparently superior treatment, for reasons of consistency, should participants already on the inferior treatment not be moved across to the better treatment (if doing so is clinically feasible)? Clearly, doing so would undermine the scientific rigour of the trial, effectively reducing it to a cohort study, but not doing so would mean that participants are maintained on the apparently inferior treatment for the sake of science, rather than to uphold individual ethics.

A reading of equipoise based on E1 seems to create important challenges for OAR. Having determined that trial data showing differential treatment effectiveness disturb equipoise, there is no clear means by which equipoise can be restored or its loss appropriately compensated for. Many of these challenges do not arise under E2, owing to its ability to maintain equipoise in the face of data that appear to favour one treatment over another. How participant welfare is regarded in RCTs based on OAR therefore depends significantly on how equipoise is construed.

### Protecting participant autonomy

Like equipoise, consent is commonly regarded as an ethical prerequisite for RCTs, as a means of upholding participants’ autonomy. However, the moral force of consent depends on its being adequately informed, as lack of information prevents meaningful choice and is thus a constraint on autonomy [51]. More specifically, in order to support autonomous choice, consent requires an appropriate equilibrium between disclosure and understanding. What potential participants are told should be sufficient to provide them with a sound factual basis for their decision, but should not be so detailed as to create confusion or information overload.

There is, however, considerable empirical evidence that the understanding and recall required for consent to be informed are very hard to achieve [52, 53]. Satisfying these requirements of understanding and recall is likely to be particularly challenging when seeking to explain a method of allocation that adapts itself dynamically during the course of the trial. Also, as Saxman indicates [9], participants need to understand that although accumulating data can cause randomization probabilities to change, they may still be allocated to the currently disfavoured treatment. Equally difficult may be explaining that the information on treatment response that causes changes to the allocation process at a particular time is only provisional, based on emerging trends, and that the definitive conclusion at the end of the trial may be different. Additionally, participants should understand that, owing to the small amount of data available, there is little on which to base the adaptive allocation to treatment arms for the first few individuals enrolled in the study, whereas there is progressively more information on which to base allocation for later participants.^12^ It is likely, therefore, that the necessary balance between disclosure and understanding will be hard to achieve; the complexity of the information required to permit an informed choice is likely to exceed many participants’ comprehension. Furthermore, this complexity may increase the likelihood of framing effects—cognitive biases whereby subtly different ways of presenting equivalent information may result in different choices [54]. These effects may weaken the validity of consent [55]. If, as a result of the potential difficulties outlined above,^13^ understanding on the part of the participant is inadequately achieved, consent loses much of its moral authority.

Furthermore, because allocation is based on a continuous re-appraisal of outcome data, information given to participants at the point of recruitment should be constantly adjusted to reflect the most recent appraisal. Likewise, updated information should be provided to those already in the trial, as the information on which they based their original consent may now be outdated. Accordingly, not only should the details given to the first few individuals to enrol differ considerably from those given to individuals enrolling at a much later point in the study, but the latter information should also be provided to those already in the trial so that their continued consent can be affirmed. If accruing information is sufficiently persuasive to adjust randomization it is presumably sufficiently persuasive to be conveyed to both new and existing participants.

These difficulties related to consent do not directly undermine the appropriateness of OAR as a research design; but given that adequate understanding is a prerequisite for consent and that consent is, in turn, a necessary condition prima facie for a trial’s being morally justified, they are challenges that must be addressed.^14^

### The therapeutic misconception

An issue with important implications for consent is the so-called therapeutic misconception [56]. This term denotes the tendency for participants to conflate clinical research and clinical practice—despite receiving detailed information clarifying the study’s scientific nature—thereby assuming that the treatment they receive in a trial will reflect their individual clinical needs, rather than the scientific goal of the study. In particular, the fact that treatment is determined by randomization, rather than clinical indication, may be misunderstood, and such misunderstanding undermines the adequacy of consent [57, 58]. Closely allied to this is the notion of therapeutic misestimation—a tendency to overestimate the benefits, or underestimate the harms, associated with trial participation [59].

Meurer et al. suggest that use of OAR may offset the therapeutic misconception by serving to ‘close the gap between what trial participants believe and what they experience’ [60, p. 2377]. In support of this claim, they point to the increasing probability that participants who join the trial later will receive the treatment ultimately found to be superior. However, given that the therapeutic misconception centres on the issue of individualized care, in order to show that the design of a trial mitigates this misconception, one would need to demonstrate that such a design adapts allocation to the individual participant’s clinical needs. This does not happen with OAR, which seeks only to adjust allocation in terms of aggregate treatment effectiveness. It remains a stochastic method of allocation, in which randomization probabilities are normally adjusted in relation to sequences of patients [61], rather than from one patient to the next, and it therefore does not align the allocation of trial interventions with the characteristics of particular individuals.

If anything, OAR is liable to reinforce, rather than alleviate, the therapeutic misconception. Such reinforcement may happen in one of two ways. First, an explanation of the process by which allocation is adjusted is likely to further reduce potential participants’ understanding that such allocation is still a random process, albeit weighted. Second, the indication that treatment allocation is influenced by evidence of differential clinical benefit may encourage participants to believe that treatment within an OAR trial will be tailored to the individual; they may mistake a change in allocation intended to favour participants in general for one directed at their specific clinical needs. Moreover, Hey and Kimmelman point out that the problem is likely to be most acute among participants allocated to the seemingly inferior arm of the study, as their allocation is most at variance with what they would expect under the therapeutic misconception [2]. Furthermore, apart from its effects on the therapeutic misconception, OAR may also encourage therapeutic misestimation. Having understood the notion that changing randomization ratios track emerging provisional evidence of therapeutic benefit, participants may attach undue weight to this fact, overlooking the provisional nature of such evidence and assuming that one’s having been randomized to the apparently superior treatment—given data collected thus far—is a strong, or even conclusive, indication that this is indeed the best intervention. In this way, while those randomized to the worse-performing arm may be particularly susceptible to the therapeutic misconception, those randomized in the other direction may be particularly susceptible to therapeutic misestimation.

As noted earlier, in some circumstances, more participants in total may be allocated to the inferior treatment than to the superior treatment, given the conclusion reached at the end of the study. Even if this situation does not characterize the trial as a whole, it may hold at a particular time within the trial’s duration—that is, at one or more points in the trial, OAR may favour the treatment ultimately shown to be inferior, even though randomization across the whole trial may prove to be weighted towards the superior treatment. In such cases, certain participants will have been randomized on what turns out to be unreliable information. The moral objection here is not that these patients should not have been allocated in this way at that time, as it is only in hindsight that the accuracy of allocation can be judged; rather, the objection is that the situation is likely to run counter to participants’ expectations, creating an additional form of misconception. While participants may understand that OAR randomizes preferentially in accordance with emerging evidence on treatment benefit, it is far less likely that they will fully appreciate the reality that such allocation may turn out to be in the ‘wrong’ direction. They are likely to have given their consent on the assumption that randomization would be weighted towards the better treatment throughout.

### Scientific validity of the trial

Although the primary importance of consent is clearly ethical, relating to considerations of autonomy, the information provided as the basis for consent and the nature of the consent process may also have implications for the validity of the trial. These methodological considerations will, in turn, have ethical implications insofar as scientific rigour is a necessary (though not a sufficient) condition for a study’s ethical justification [62]. If consent requires that participants be informed about the weighting of randomization at the time of enrolment, then those who subsequently discover that they are in the disfavoured treatment arm may be more likely than other participants to drop out of the study [5], thereby undermining the statistical comparability of the treatment groups. Similarly, if early enrollees should be informed of later changes in the weighting of randomization, as I argue above, then resentful demoralization or compensatory rivalry may ensue amongst those who find themselves in the disfavoured treatment arm, with a consequent biasing of outcomes.^15^ Further, obliging researchers to update participants on changes in allocation probabilities would likely preclude participant blinding in trials where treatment concealment is important. Similarly, the trialist would be unblinded as to the relative performance of the treatments being tested [10]. This lack of blinding could lead to bias, either in participants’ responses to treatment or in individual investigators’ recruitment behaviour or assessment of outcomes [64].

## Conclusions

**Fig. 1:**
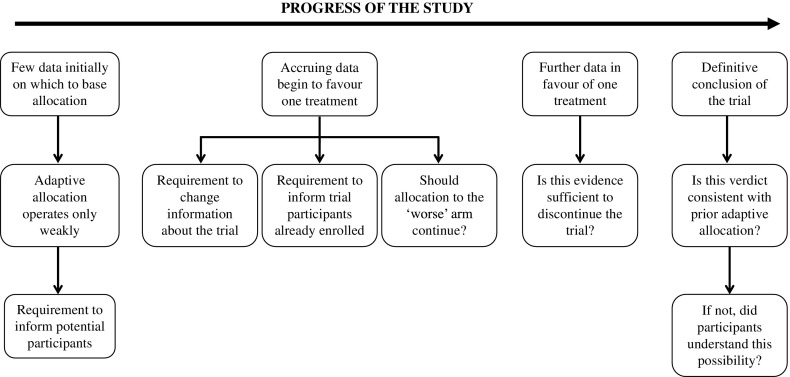
Ethical issues arising during the progress of a trial with outcome-adaptive randomization

OAR gives rise to several ethical issues related to welfare and autonomy, and these issues are largely connected to the way in which OAR responds to changing information during the trial (Fig. 1). Of course, if the null hypothesis is ultimately retained, then there is no ‘better’ or ‘worse’ treatment, and whichever arm participants were allocated to during the trial may seem inconsequential [6]. However, while such an outcome may obviate the problem of inappropriately weighted randomization, it also removes the intended benefit of OAR.

At the root of welfare-related issues in OAR is the notion of equipoise. If the advocate of OAR adopts the first model of equipoise that I have described, E1, then these issues are acute, as he or she is committed to regarding an intervention disfavoured by emerging data as inferior and thus as a threat to participant welfare. For an advocate of OAR who subscribes to E2, however, a disfavoured intervention retains legitimacy provided it is still recommended by some portion of the expert clinical community.

It would appear that OAR does not uphold—and therefore cannot appeal to—the notion of individual ethics, as allocation does not respond to the individual characteristics or needs of each participant. Instead, OAR seems to rely more on collective than on individual ethics, focusing on the idea that, in aggregate, more patients will be allocated to the better treatment. Thus, when Daryl Pullman and Xikui Wang argue that OAR seeks to ‘treat as many patients as effectively or successfully as possible’ [18, p. 204], they retreat from individual to collective ethics. Unfortunately, OAR may not fare well once viewed in terms of collective ethics: although the proportion of patients allocated to the better treatment under OAR is greater than under FRR, the number of such patients may not be—and when choosing between these two designs, it is surely the number, not the proportion, that should feature in the consequentialist balancing of benefit and harm that lies at the heart of collective ethics. Thus, the claim that OAR protects individual ethics in the context of RCTs appears to be unfounded. Instead, much of the ethical rationale for OAR is centred in collective ethics, and upon entering that territory, it appears to fare worse than FRR. In fact, it can be argued more generally that the pursuit of individual over collective ethics is misplaced in clinical trials. The purpose of such studies is to generate valid conclusions as to aggregate treatment effectiveness, and this requires individual clinical decision-making to be at least partly subordinated to the demands of the research design—as the therapeutic misconception indicates. In the final analysis, clinical trials are concerned with reaching a decision about patients as collectivities rather than as individuals. Consequently, with some exceptions (e.g., monitoring for adverse events in individual participants or ensuring that consent is still in place), ethical concern is with the collective welfare of participants in the study, not with the welfare of each participant taken individually. Pursuing the latter—such as by trying to allocate each participant according to his or her specific clinical presentation rather than by a wholly random mechanism—is likely to run counter to the methodological demands of the study. This is not to deny that patients may benefit by participating in clinical trials [65], but it indicates that such benefits are not individuated.

Trials based on OAR raise questions regarding the different prospects of benefit for early versus late enrollees and regarding the way in which emerging information seems to determine the handling of new recruits but not participants already in the study. In addition, while clear empirical evidence may be lacking, it is reasonable to think that OAR presents considerable challenges for disclosure on the part of the researcher and for comprehension on the part of the participant. Particular problems in this regard centre on the notions of therapeutic misconception and therapeutic misestimation.

Some of the concerns that have been outlined in respect of OAR can be mitigated by design modifications. For example, the likelihood of weighting randomization to the ‘wrong’ treatment can be reduced by restricting the range of randomization probabilities or by employing an initial burn-in with equal randomization [50, 66]; and using baseline information through a more elaborate process of covariate-adaptive response-adaptive randomization might bring treatment allocation closer to the individual patient [67, 68].^16^ However, there remain other ethical difficulties with OAR that are less amenable to reparative strategies at the level of design.

### Does fixed-ratio randomization fare better?

The advocate of OAR—or, at least, one who subscribes to E1—might argue that FRR fares no better. One criticism might be that by taking no account of accumulating data on treatment effectiveness, other than at specific interim analyses, FRR simply ignores information relevant to participants’ welfare [7]. Worse yet, so the objection might run, the FRR trialist is prepared to randomize 50% of participants to the seemingly inferior treatment in the face of such information, whereas OAR strives to randomize fewer. Thus, Palmer contends that ‘possible 9:1 randomization in adaptive designs … remains a better deal for participants than 1:1 randomization’ [47, p. 393], and Pullman and Wang argue that the last patient enrolled in a trial employing FRR has only a 50% chance of receiving the better treatment, though this chance is much higher for the first patient treated after completion of the trial [18, p. 208]. One response on behalf of FRR could be that no account is taken of accruing, as opposed to interim, evidence because it is insufficiently informative. Stuart Pocock states that the principal role of interim analyses is ‘to look for treatment differences which are sufficiently convincing and important to stop or change the trial’ [24, p. 143]. On this basis, it might be argued that accruing data, assessed *pari passu* with participant allocation, do not constitute ‘convincing’ evidence and therefore do not substantiate any claim that participants have received, or failed to receive, the better treatment during the course of the trial; such evidence may be obtained only through a formal statistical evaluation at a prespecified interim analysis.^17^ As a second rejoinder, advocates of interim analyses in the context of FRR might claim to respond more fully to a loss of equipoise by halting the trial or perhaps dropping a treatment group, in contrast to the somewhat partial and inconsistent response in the context of OAR.

Donald Berry defends OAR against charges of inefficiency by indicating that in some cases where there are both safety and efficacy objectives, a randomization ratio of 4:1 may be more efficient, in terms of the required number of participants, than a ratio of 1:1 [3]. However, this argument appears to be one favouring unequal over equal randomization in such circumstances rather than one favouring OAR over FRR. Advocates of FRR need not insist on 1:1 randomization; they would simply require that if imbalances in treatment arms brought about by OAR reduce efficiency, this should be justified by countervailing ethical considerations.

With regard to consent, both OAR and FRR face challenges in the way of achieving appropriate disclosure and comprehension, particularly in relation to randomization. However, even if these objectives are imperfectly met in FRR, they are probably achieved to a lesser extent in OAR. A straightforward process of randomization is likely to be easier to explain and understand than one framed in terms of changing probabilities of allocation, and difficulties with the therapeutic misconception or therapeutic misestimation are likely to be more acute in OAR.

Overall, and depending in part on the construal of equipoise that underlies its use, a moral case for OAR in terms of welfare and autonomy has yet to be established.

## Footnotes

1 I follow Spencer Hey and Jonathan Kimmelman’s [2] landmark paper in focusing on the two-arm case. It has been argued by some [14–17] that in terms of certain methodological and statistical characteristics, OAR has more to offer in multi-arm trials than in two-arm trials—though merits are nonetheless claimed for the two-arm case [14]. However, most of the ethical issues to be discussed apply to both the two-arm and the multi-arm context; instances in which different considerations may apply will be noted.

2 This translates as: An experimental design or strategy is based on collective ethics if it conduces to maximizing the total benefit of the group, and conversely it is founded on individual ethics if it conduces to maximizing benefit for each participant, taken individually, at the point at which treatment is to be given.

3 These are specific circumstances; as a general rule, unbalanced randomization leads to reduced statistical power.

4 In addition, while E2 provides a clear account of idealized changes in the size of the clinical community favouring an intervention in response to emerging evidence, and of how this might provide a motivation to alter randomization weights, it is less clear how, in practical terms, these changes might translate to specific decisions to adjust these weights at the level of the trial. A more developed account of how this might occur is needed for a full evaluation of E2 in the context of OAR.

5 It should be noted that E2, as expounded by London [11], rejects such a notion of ‘belief’, since it suggests an epistemologically implausible model of a single meta-agent whose beliefs regarding emerging data are required to be reconciled. Instead, evidence emerging from the trial is taken by London to represent a distribution of beliefs within an idealized medical community.

6 Thus, Jason Tehranisa and William Meurer argue that OAR ‘works to *collectively* favour the patients within the trial in situations when one treatment is ultimately better than the other’ [39, p. 2131] (emphasis added).

7 The proviso ‘may be greater’ is stated because, depending on both the size of the study and the proportion of participants assigned to the ultimately inferior treatment, there may be certain instances where the number receiving the inferior treatment under OAR is smaller than would be expected with 1:1 FRR, though the difference is likely to be modest [43]. Similarly, the total number of patients required in an OAR study may in certain circumstances be smaller than the number required in an FFR trial [42].

8 There is, however, evidence that under certain conditions multi-arm trials employing OAR may be more efficient than two-arm OAR trials, and sometimes more efficient than a multi-arm trial with FFR [15, 16].

9 Within perfect procedural justice, a process of allocation can be defined that will guarantee a just substantive outcome (where such an outcome is defined in terms other than the allocation process per se). Within imperfect procedural justice, a just substantive outcome is not guaranteed, as ‘there is no feasible procedure which is sure to lead to it’ [45, p. 86].

10 If such choice as to the timing of enrolment were in fact feasible, it would raise issues for the internal validity of the study, as the changing randomization ratios would tend to be associated with changes in the characteristics of the participants being allocated [9, 10].

11 Hey and Kimmelman discuss this issue with specific reference to phase 2 and phase 3 clinical trials [2]; see also Jack Lee [7]. The possibility that the criterion for adaptive randomization might be based on more than one outcome variable presents a challenge. In covariate-adaptive randomization, baseline covariates can be differentially weighted in terms of their potential confounding influences—which might be determined empirically—and the randomization algorithm can be determined accordingly. In OAR, however, such outcomes would have to be weighted in terms of their relationship to a particular conceptualization of participant welfare. Determining the appropriate weightings in such terms would not be straightforward.

12 As is indicated in earlier discussion, the role that such information plays will differ according to whether E1 or E2 is adopted as the underlying criterion of adaptive randomization. In either case, however, the requirement to tell participants that—and how—such information is to be used is essentially the same.

13 There is little empirical evidence in the specific context of OAR, but these assumptions seem reasonable given what is known about information and recall in relation to consent. One experimental study that has examined comprehension of OAR found that, while self-rated understanding of OAR versus FRR did not differ, correct identification of the method of treatment allocation was significantly lower for those presented with a description of OAR than for those presented with a description of FRR [39]. It is also reasonable to think that challenges in terms of disclosure and comprehension are greater in a multi-arm than in a two-arm OAR trial.

14 Steven Joffe and Susan Ellenberg [5] as well as Bothwell and Kesselheim [12] consider some practical issues relating to obtaining consent with OAR.

15 Resentful demoralization describes a phenomenon whereby those perceiving themselves to be recipients of the less desirable intervention may become disheartened and respond less well. In contrast, compensatory rivalry occurs when such individuals respond better in an attempt to offset the perceived disadvantage of receiving the less favourable intervention [63]. An additional possibility is switching—those aware that they are receiving the less desirable intervention may try to obtain the better alternative.

16 Some design modifications that aim to strengthen OAR trials have associated disadvantages. Greater efficiency in a multi-arm OAR trial may be gained by maintaining the size of the control group at that of the best-performing active treatment arm. However, this modification would undercut the ethical motivation of OAR by preventing preferential randomization to this arm in comparison to the control arm [10].

17 This counterargument depends, however, on the appropriate number and timing of interim analyses. If such analyses are too few, or occur too infrequently, or adopt an unsuitable statistical threshold for termination of the trial [69, 70], there is a sense in which a trial based on FRR would indeed be open to the charge of taking inadequate account of relevant information.
